# Increased professionalization and lower burnout scores were associated with structured residency training program: results of a cross sectional survey

**DOI:** 10.1080/10872981.2021.1959284

**Published:** 2021-07-29

**Authors:** Michaela Olm, Marco Roos, Alexander Hapfelmeier, Dagmar Schneider, Jochen Gensichen, Pascal O. Berberat, Antonius Schneider

**Affiliations:** a TUM School of Medicine, Institute of General Practice and Health Services Research, Technical University of Munich, Munich, Germany; b Kompetenzzentrum Weiterbildung Allgemeinmedizin Bayern (KWAB), Erlangen, Germany; c Institute of General Practice, Friedrich-Alexander University Erlangen-Nürnberg (FAU), Erlangen, Germany; d TUM School of Medicine, Institute of Medical Informatics, Statistics and Epidemiology, Technical University Munich, Munich, Germany; e Koordinierungsstelle Allgemeinmedizin, Munich, Germany; f Institute of General Practice and Family Medicine, University Hospital of the Ludwig-Maximilians University of Munich, Munich, Germany; g TUM Medical Education Center, School of Medicine, Technical University of Munich, Munich, Germany

**Keywords:** Family medicine, burnout, professionalization, resident physicians, vocational training

## Abstract

The competence centre for Residency Training in Family Medicine Bavaria (CCRTB) was established to improve the quality of postgraduate medical education by offering training and mentoring programmes for residents, and by providing train-the-trainer and mentoring courses for supervisors. Beyond that, regional Residency Training Networks (RTN) on avoluntary basis were developed to facilitate structured and efficient clinical rotation programs. Primary aim was to investigate the burden of burnout and the development of professionalism among CCRTB-residencies within a cross-sectional study. Secondary aim was to evaluate differences between CCRTB-residents with and without participation in aregional RTN. Burnout was determined with the Maslach Burnout Inventory (MBI), comprising the scales emotional exhaustion, depersonalization, and personal accomplishment. Ambulatory professionalization was evaluated using the German Professional Scale (Pro-D), comprising the scales professionalism towards the patient, towards other professionals, towards society, and towards oneself. Statistical significance of group differences was calculated by nonparametric tests. Multivariable linear regression modelling was performed to estimate the independent impact of professionalization and RTN participation on burnout scores. 347 CRRTB residents in ambulatory postgraduate training were invited, 212 (61.1%) participated, and 197 (92.9%) were included in our analyses. Lower emotional exhaustion and depersonalization, and increased personal accomplishment was associated with increased professionalisation, which was significant for nearly all Pro-D scales (p ≤ 0.05). RTN residents showed higher professionalism towards the patient (p = 0.031), other professionals (p = 0.012), and towards the society (p = 0.007) than residents of unstructured programs, and higher levels of personal accomplishment (p < 0.05). Early and efficient professionalization might be akey to reduce burnout and to establish asatisfying career in family medicine. Train-the-trainer and mentoring concepts should be implemented regularly for the training of residents. Thus, increased engagement in medical didactics should be aprerequisite for accreditation as atraining practice for residents.

## Introduction

According to Maslach et al. burnout is defined as a continuous construct characterized by emotional exhaustion, depersonalization and reduced personal accomplishment [1]. Physicians’ burnout represents a special matter of concern because burnout is more common among physicians than in the general population [2] . This psychological burden is already visible during medical school and residency. A systematic review of Ishak et al. identified a burnout prevalence of 28–45% in medical students and 27–75% in medical residents [3]. The phase of postgraduate medical training represents a peak time of distress [4] and a new level of personal involvement has to be solved [5]. Sudden assumption of responsibility, fear of showing imperfection in front of supervisors, lack of recognition from senior doctors, the interaction with the medical team or patients [5], as well as a high workload, an insufficient work-life balance and on-call duties can be stressors [6,7]. Burnout is also an important issue for family practice [8]. Less control over workload, small scope of practice, management, and budget-related responsibilities might contribute to workplace distress specifically to family practice [9–11]. Focusing on trainees in family medicine, a prevalence of 36% was found [12]. The adverse effects of leaving the issue of burnout unaddressed are medical errors and poorer health outcomes [13]. Beyond that, increased burnout may make physicians less willing to start their own family practice. In this context, it is of particular importance that mentoring and a supportive learning environment can be approaches to reduce burnout in medical residents [14,15].

In Germany, postgraduate medical specialization for family medicine residents (FMR) lasts at least 5 years. Although the state medical boards in Germany have detailed regulations for postgraduate medical training, the residency training is structurally and educationally disorganized. Clinical rotations are generally not tied to training programs or academic centres. During this time, 24 months have to be completed in family practice. In addition, 12 months must be completed in the field of internal medicine in acute inpatient care. Furthermore, 6 months have to be completed in at least one other area of direct patient care. Following a modular concept, up to 18 months of continuing education in other areas of direct patient care must then be completed. Normally, FMRs organize the individual units of their residency training themselves. This is disadvantageous due to inefficient timing and organizational challenges, since, for example, new employment contracts have to be concluded for each new section. These organizational constraints lead to an extension of the further training period by up to 8 years for male and 10 years for female physicians [16].

FMR in Bavaria, Germany, has the possibility to enroll in the ‘Competence Centre for Residency Training in Family Medicine Bavaria’ (CCRTB) (German: Kompetenzzentrum Weiterbildung Allgemeinmedizin Bayern, KWAB) since 2017 [17]. The competence centres were implemented in all federal states of Germany to improve the educational situation of the FMR. The aim of a postgraduate training in the CCRTB is a structural and professional organisation of educational training in family medicine, in order to reach a higher level of coordination and quality [18]. The enrolment in the CCRTB is free, without competition, and on a voluntary basis for the FMR. The development of professionalism, one of the seven required core competencies of family medicine, plays a central role and should be promoted by establishing a competence-based curriculum [19,20]. Therefore, the CCRTB network offers specific didactic courses and mentoring programs for trainees, and also train-the-trainer courses for supervising physicians on a voluntary basis. These criteria were established as quality indicators for good postgraduate training [18,21].

In addition to their enrolment in the CCRTB, residents have the opportunity to participate in a regional residents training network (RTN) on voluntary basis. The regional RTN was implemented to foster residency training in family medicine. A regional RTN consists of hospitals and medical practices with a structured organisation to enable a scheduled and efficient clinical rotation of the residents within the various clinical wards and practices [22]. The regional RTN was established on the basis of local initiatives in cooperation with the CCRTB to enable a time-efficient and didactically attractive residency training. In this context, an appealing RTN concept is intended to motivate young physicians for residency training in areas with a shortage of physicians. Therefore, the liabilities for regional RTN are more distinct because they are characterized by binding commitments with regard to rotation options, contract duration, and time-offs for medical education events. Beyond that, the medico-didactic concepts are intensified within the regional RTN. For example, the supervising physicians of an RTN are more committed to participate in medico-didactic courses, train-the-trainer and mentoring seminars of the CCRTB to ensure high quality in education and to enable a fast professionalization of the FMR [18]. The FMR participate in regional RTN on a voluntary basis without competitive selection. The primary aim of the study was to investigate the burden of burnout and the development of ambulatory professionalism among postgraduate FMR enrolled in the competence centre. The secondary aim was to evaluate differences between CCRTB-residents with and without participation in a regional RTN.

## Methods

### Study design

We performed a cross-sectional study. All residents in family medicine training that were enrolled in the Bavarian CCRTB were personally invited by email to participate in our study and to complete an online self-report survey on the internet-based platform LimeSurvey®. The reminder was sent out four times by email every 3 weeks. If residents had not completed the survey after the fourth reminder, a paper-pencil version of the questionnaire was sent by post. Again, all non-respondents received a postal reminder after 5 weeks.

### Questionnaires

To assess the amount of resident’s burnout, we used the Maslach Burnout Inventory Human Services Survey for Medical Personnel (MBI-HSS MP) [23]. The MBI-HSS MP contains 22 items, which are aggregated in three subscales: 1) the emotional exhaustion (EE) scale measures feelings of being emotionally overextended and exhausted by one’s work (9 items; range 0–54), 2) the depersonalization (DP) scale measures an unfeeling and impersonal response toward recipients of one’s care or treatment (5 items; range 0–30), and 3) the personal accomplishment (PA) scale measures feelings of competence and successful achievement in one’s work (8 items; range 0–48). Scores of the online survey are recorded on a 7-point Likert scale from ‘Never’ (0) to ‘Every day’ (6). Higher scores on EE and DP and lower scores on PA indicate a higher risk of burnout. We calculated the sum scores for each dimension, using the following cut-off values according to Maslach et al. [23]:

- EE: ≤18 low burnout level; 19–26 average burnout level; ≥27 high burnout level
- DP: ≤5 low burnout level; 6–9 average burnout level; ≥10 high burnout level
- PA: ≥40 low burnout level; 39–34 average burnout level; ≤33 high burnout level

For the assessment of ambulatory professionalization, we used the German Professional Scale (Pro-D) [24], a German adaptation of the Nijmegen Professional Scale [25]. The Pro-D consists of 67 items, which are aggregated in four subscales: 1) professionalism towards the patient (21 items; range 21–84), 2) professionalism towards other professionals (14 items; range 14–56), 3) professionalism towards society (10 items; range 10–40), and 4) professionalism towards oneself (22 items; range 22–88) (see Supplement, Table S 5). According to Tromp et al. [25], scores are recorded on a 4-point Likert scale from ‘seldom’ (1) to ‘always’ (4). A higher sum score represents a higher level of professionalization. The Pro-D showed good internal consistencies with Cronbach's alpha coeficients of the scales ranging from 0.79 to 0.95 [25].

Beyond that, age, gender, year of postgraduate training, working hours per week, and participation in a regional RTN were documented.

### Data analyses

We performed descriptive analyses (means, standard deviations, and frequencies) for the sociodemographic data (age, gender, participation in a regional RTN, total number of hours spent working per week, and MBI subscales) of the included residents. In addition, hypothesis testing of the differences between gender, participation in a regional RTN, and years of training in terms of ‘burnout’, as well as differences between gender, participation in a regional RTN, and years of training was performed by non-parametric tests. For dichotomous characteristics (gender and participation in a regional RTN) we applied the Mann–Whitney U-test, for ‘years of training’ the Kruskal-Wallis-test was used. Multivariable linear regression modelling was performed to estimate the independent effect of ambulatory professionalism, years of training, and participation in a regional RTN on the degree of burnout symptoms.

Due to a transmission error, the postal survey was carried out with a 6-point scale (missing category 5: ’A few times a week’). To achieve an approximation of online and postal results, answers in category 4 of the postal questionnaires were replaced by the mean values of 4 and 5 of the online questionnaires. In order to assess the amount of possible bias, sensitivity analyses without postal questionnaires were performed.

All data were analysed with SPSS version 25 (IBM Corp., New York, USA). Questionnaires with >50% missing answers were excluded from the analyses. Single missing items (≤20% within a scale) were imputed using the scale mean. Overall, a level of significance of p < 0.05 was used. The study was approved by the Ethics Committee of the Medical Faculty of the Technical University Munich (161/19 S). Participation in this study was voluntary and all family medical residents were informed in detail about the study's aims. In addition, anonymity and confidentiality regarding the data were granted. Informed consent was obtained from all participants.

## Results

### Sample

According to the annual balance, a total of 1720 FMRs in family practice were registered in Bavaria in 2019. Three hundred and forty-seven (27.3%) FMR were enrolled in the CCRBT in May 2019; 288 (83.0%) were female, the mean age was 37.4 years. They were invited to participate via email. Two hundred and twelve (61.1%) participated. One hundred and ninety-seven (92.9% of 212) FMR were included into the analysis, 15 (7.1% of 212) were not included due to missing data. One hundred and sixty-six participants (84.3% of 197) were female, mean age was 37.2 years (Table 1). Forty-one (20.8%) participated in a regional RTN (11 (5.6%) data missing). There were no significant differences between responder and non-responder; the mean age of non-responders was 37.7 years (p = 0.52), 135 (78.9%) were female (p = 0.08), and 36 (20.0%) participated in a regional RTN (p = 0.67) (not in Table).

**Table 1.** Characteristics of the included ambulatory residents

Total sample (N, (%))		197	(100)
Gender: female (N,(%))		166	(84.3)
Age (M, (SD))		37.2	(7.0)
Participation in a regional RTN (N, (%))		41	(20.8)
Working hours per week (M, (SD))		32.5	(8.8)
Year of training			
1.		6	(3.0)
2.		17	(8.6)
3.		29	(14.7)
4.		66	(33.5)
≥5.		73	(37.1)
Degree of burnout			
Emotional Exhaustion (N, (%))			
Low		107	(54.3)
Average		49	(24.9)
High		39	(19.8)
Depersonalization (N, (%))			
Low		113	(57.4)
Average		43	(21.8)
High		40	(20.3)
Personal Accomplishment (N, (%))			
Low		116	(58.9)
Average		48	(24.4)
High		23	(11.7)

M: mean; N: number; RTN: residents training network; SD: standard deviation.

Thirty-nine (19.8% of 197) of the residents showed high burnout scores regarding emotional exhaustion, 40 (20.3%) in terms of depersonalization, and 23 (11.7%) in terms of personal accomplishment (Table 1). The highest emotional exhaustion, depersonalization and lowest personal accomplishment scores were observed within the first year (Table 2). This effect attenuates over the years of training. However, the differences were not significant. Men showed higher scoring in the depersonalization scale (p = 0.006). There were no significant differences between RTN participants and the other residents.

**Table 2.** Burnout symptoms (mean values of emotional exhaustion, depersonalization, personal accomplishment), differentiated by gender, participation in a regional RTN, training phase, and year of training

	Emotional exhaustion			Depersonalization			Personal accomplishment		
	N	M	(SD)	N	M	(SD)	N	M	(SD)
**Total**	195	18.7	(9.6)	196	6.0	(4.9)	187	40.2	(6.1)
*Gender*^a^									
Female	164	18.4	(9.5)	165	5.5	(4.6)	157	40.2	(6.0)
Male	29	20.0	(10.4)	29	8.4	(5.8)	28	39.6	(7.1)
		*p = 0.375*			*p = 0.006*			*p = 0.887*	
*Year of training*^b^									
1.	5	28.8	(7.6)	6	8.3	(7.4)	4	36.5	(5.2)
2.	17	17.5	(8.0)	17	6.2	(5.1)	15	39.7	(5.4)
3.	29	17.1	(9.3)	29	5.9	(5.1)	28	40.1	(6.0)
4.	65	19.1	(9.6)	66	5.9	(4.5)	64	41.3	(4.9)
≥5.	73	18.8	(10.2)	72	5.7	(5.1)	71	39.4	(7.3)
		*p = 0.129*			*p = 0.862*			*p = 0.351*	
*Participation in a regional RTN*^a^									
Yes	41	20.1	(12.1)	41	6.4	(5.7)	38	38.6	(7.0)
No	145	18.5	(8.9)	146	5.9	(4.7)	141	40.5	(6.0)
		*p = 0.702*			*p = 0.932*			*p = 0.129*	

^a^Test of significance: Mann–Whitney U-test; ^b^Test of significance: Kruskal-Wallis-test; Level of significance: p < 0.05; M: mean; N: number; RTN: residents training network; SD: standard deviation. Cut-off values for ‘high’ burnout as suggested by Maslach et al. [22]: emotional exhaustion: ≥27; depersonalization: ≥10; personal accomplishment: ≤33.

Residents of regional RTN showed significantly higher scores in the professionalism scales towards the patient (p = 0.031), towards other professionals (p = 0.012), and towards society (p = 0.007) (Table 3). Females showed a higher professionalism towards the patient (p = 0.039). Professionalism towards the patients increased significantly with increasing years of training (p = 0.040).

**Table 3.** Degree of professionalism (sum score), differentiated by gender, participation in a regional network, and year of training

	Professionalism towards the patient			Professionalism towards other professionals			Professionalism towards society			Professionalism towards oneself		
	N	M	(SD)	N	M	(SD)	N	M	(SD)	N	M	(SD)
**Total**	197	72.7	(5.55)	177	44.3	(5.27)	187	31.2	(4.17)	196	71.5	(6.77)
*Participation in a regional RTN*^a^												
Yes	41	74.5	(5.08)	38	46.1	(4.53)	40	32.7	(4.11)	41	72.5	(8.08)
No	147	72.4	(5.56)	131	43.8	(5.38)	138	30.7	(4.13)	146	71.3	(6.53)
		*p = 0.031*			*p = 0.012*			*p = 0.007*			*p = 0.232*	
*Gender*^a^												
Female	166	73.1	(5.32)	149	44.4	(5.30)	158	31.1	(4.26)	165	71.8	(6.54)
Male	29	70.6	(6.51)	26	43.2	(5.23)	27	31.4	(3.82)	29	69.6	(8.02)
		*p = 0.039*			*p = 0.259*			*p = 0.794*			*p = 0.255*	
*Year of training*												
1.	6	68.1	(3.54)	5	41.4	(3.16)	5	30.9	(2.51)	5	64.4	(5.86)
2.	17	71.0	(5.19)	12	42.7	(5.60)	15	29.4	(5.48)	17	69.7	(5.97)
3.	29	71.9	(6.24)	28	43.7	(5.33)	27	30.5	(3.97)	29	72.4	(6.08)
4.	66	73.2	(5.15)	58	44.3	(5.38)	63	31.2	(4.10)	66	71.8	(6.73)
≥5.	73	73.8	(5.33)	68	45.4	(5.02)	71	32.0	(4.02)	73	72.2	(6.69)
		*p = 0.040*			*p = 0.207*			*p = 0.369*			*p = 0.071*	

^a^Test of significance: Mann–Whitney U-test; ^b^Test of significance: Kruskal-Wallis-test; Level of significance: p < 0.05; M: mean; N: number; RTN: residents training network; SD: standard deviation

The multivariable linear regression model showed that lower emotional exhaustion was associated with increased professionalization, which was significant for all scales of the Pro-D (p ≤ 0.05) (Table 4). Lower depersonalization was associated with higher professionalism towards the patient (p = 0.003), towards other professionals (p = 0.048), and professionalism towards oneself (p < 0.001). Increased personal accomplishments were significantly associated with all scales of the Pro-D (p < 0.01). The years of training showed no significant effect in the regression model. The participation in a regional RTN had a significant positive influence on personal accomplishment with respect to all scales of the Pro-D (p < 0.05).

**Table 4.** Multivariable linear regression modelling of professionalism, years of training, and participation in a regional network. Outcome: Burnout sums up scores of emotional exhaustion, depersonalization, and personal accomplishment

**Explanatory variable**	**Emotional exhaustion**			**Depersonalization**			**Personal accomplishment**		
	**β**	**SD**	**p value**	**β**	**SD**	**p value**	**β**	**SD**	**p value**
Constant	47.082	10.842		22.446	5.422		13.311	6.863	
Professionalism towards the patient	−0.337	0.138	0.015	−0.207	0.069	0.003	0.319	0.087	0.000
Year of training	0.029	0.695	0.967	−0.018	0.345	0.958	−0.246	0.449	0.585
Participation in a regional RTN	−2.052	1.753	0.243	−0.708	0.881	0.423	2.492	1.116	0.027
Constant	35.825	8.310		14.330	4.186		24.032	5.310	
Professionalism towards other professionals	−0.304	0.153	0.049	−0.153	0.077	0.048	0.272	0.096	0.005
Year of training	−0.113	0.757	0.882	−0.225	0.381	0.555	−0.282	0.488	0.565
Participation in a regional RTN	−1.688	1.883	0.372	−0.374	0.946	0.693	2.830	1.194	0.019
Constant	33.816	7.351		11.306	3.708		25.516	4.581	
Professionalism towards society	−0.363	0.184	0.050	−0.125	0.093	0.180	0.349	0.114	0.003
Year of training	−0.052	0.720	0.943	−0.174	0.364	0.634	−0.301	0.464	0.518
Participation in a regional RTN	−1.968	1.816	0.280	−0.483	0.916	0.599	2.817	1.145	0.015
Constant	49.906	8.498		23.500	4.244		14.491	5.319	
Professionalism towards oneself	−0.390	0.106	0.000	−0.224	0.053	0.000	0.315	0.066	0.000
Year of training	0.043	0.675	0.949	−0.148	0.338	0.663	−0.211	0.435	0.628
Participation in a regional RTN	−1.785	1.704	0.296	−0.499	0.852	0.559	2.148	1.079	0.048

RTN: residents training network; SD: standard deviation; Level of significance: p < 0.05; Note: Higher values in emotional exhaustion and depersonalization, but lower values in personal accomplishment indicate a higher degree of burnout symptoms.

The sensitivity analyses showed even more pronounced results when the analyses were performed without postal questionnaires. The mean values of emotional exhaustion and depersonalization of the ´online group´ were slightly higher, and personal accomplishment slightly lower than for the whole group. Beyond that, there were significant associations between ´year of training´ and professionalism towards other professionals (p = 0.021), and professionalism towards oneself (p = 0.009) (see Supplement, Table S3). The association between ´RTN participation and personal accomplishments was stronger for the ´online group´ (p < 0.01) (see Supplement, Table S4) than for the whole group (p < 0.05) (Table 4). Details of the sensitivity analyses are depicted in the supplement (see Supplement, Table S 1–4)

## Discussion

The present study showed that FMR had moderate burnout scores in family practice, with around 20% having a critical score in the emotional exhaustion and depersonalization scales. Increased professionalization was associated with lower emotional exhaustion and depersonalization, and increased personal accomplishment. RTN residents showed higher professionalization and personal accomplishment compared with residents in unstructured training.

Several studies in different disciplines have shown increased burnout symptoms in residents [26–29]. This is also true for family medicine, especially when residents have fewer years of experience [9,30]. This is attributed to higher workplace stress, but also to contrasting expectations of their ideals of family medicine and the reality of the current workplace [9]. Compared to our results, a recently published study by Bugaj et al., which investigates the burnout in a postgraduate training programme in Baden-Württemberg, observed slightly higher degrees of burnout (EE: 33.5%; DP: 35.2%; PA: 14.6%) [31]. This might be explained by the fact that the physicians in their evaluation were younger, accompanied with lower clinical experience. Matching this, we also found the highest burnout scores during the first year of residency training. Kramer et al. described that self-perceived clinical competence of residents in family medicine training accumulates during postgraduate training [32]. Kocalevent et al. found that personal accomplishments increased continuously over time [33]; they concluded that this might act as a buffer compensating to some extent for the physicians’ stress experience. With respect to these studies, one could speculate whether ´years of training´ represent a surrogate parameter for professionalism. Fitting to this, ‘years of training’ was not significant in our regression model, whereas increased professionalization as determined with the Pro-D was significantly related with decreased burnout scores.

Previous studies have already described that burnout was associated with poorer professionalization in terms of unprofessional behaviour, e.g., suboptimal adherence to treatment guidelines, poor empathy, or dishonesty [34,35]. We identified an intense association between MBI and Pro-D scores, which might imply a protective effect of professionalization against burnout. In this context, the increased levels of professionalization and personal accomplishment of RTN residents are of particular importance. This effect might be explained by the increased engagement and cooperation between regional hospitals and family physicians, and their stronger commitment to mentorship and binding rotation concepts. Beyond that, the supervising physicians in hospitals and practices of the regional RTN might be more engaged in mentoring and participate intensively in medico-didactic trainings due to a higher intrinsic motivation. While other countries with strong primary care orientation have a longstanding tradition and elaborated concepts in FMR training [36–39], the improvement of FMR training in Germany has just begun. Our evaluation shows the effects of a training in a population that is still raw. This may alleviate to discover the relation between decreased burnout and increased professionalization. Therefore, our results underline the need to implement efficient professionalization programs on a regular, nationwide basis, similar to more ´advanced´ countries with strong primary care. Structured regional RTNs should be supported to ensure scheduled rotations and in-depth training programs. It might be valuable to evaluate within future FRM training programs, which aspects of professionalization specifically contribute to burnout prophylaxis, and how to use them to ensure a high level of job satisfaction.

### Strengths and limitations

The main strength of our investigation is the combination of burnout measurement using the Maslach Burnout Inventory [23] and the direct measurement of professionalization using the Pro-D [24], in contrast to just applying the parameter ‘years of training’. However, our study has several limitations. A limitation is that the postal survey (N = 58) of the Maslach Burnout Inventory was conducted with a 6-point scale (missing category 5: ’A few times a week’) due to a transmission error. As described in the methods section, we made an adjustment to reach a better comparability of online and postal respondents. Sensitivity analyses without postal questionnaires showed an even more pronounced effect in this group. We have chosen a more conservative approach in the analysis of our data by using all responders. However, this might have led to a slight underestimation of our results.

We performed several statistical procedures, which carry the increased risk of false-positive findings due to the multiple testing problem. However, all analyses have been performed in an exploratory, i.e., non-confirmatory, manner. In addition, we consider the risk of false negatives to be more relevant in the discussion of the present findings given the limited sample size and associated limited power of hypothesis testing. Beyond that, the results tend in the same direction, which points towards a consistency of the results. Another limitation is that only 61.1% responded to the invitation. However, we found no significant differences between responders and non-responders with respect to age, gender, or RTN participation. We had only six first-year residents, which limits the generalizability regarding stressful early years. However, these effects are consistent with previous studies. Beyond that, it has to be mentioned that we only observed residents who were enrolled in the CCRTB. One could speculate that these residents have inherently protective characteristics against burnout, such as more effective coping mechanisms or genuinely increased professionalism. Therefore, future investigations should compare residents within and outside the CCRTB with respect to different levels of burnout and professionalism. Finally, the evaluation was performed within a cross-sectional study design, which does not allow causal conclusion. Therefore, future controlled studies should apply longitudinal methods to identify possible causal relationships of the regional RTN.

## Conclusions

Early and efficient professionalization might be a key to reduce burnout and to establish a satisfying career in family medicine. However, these effects should ideally be confirmed within longitudinal controlled trials. Train-the-trainer and mentoring concepts should be implemented regularly for the training of residents. Thus, increased engagement in medical didactics should be a prerequisite for accreditation as a training practice for residents.

## Supplementary Material

Supplemental Material

## Data Availability

The data of this study are available from the corresponding author on reasonable request.

## Members of the Bavarian Competence Center for Residency Training (BCCRT) Network

Ildikó Gagyor, Jochen Gensichen, Thomas Kühlein, Marco Roos, Antonius Schneider, Dagmar Schneider, Anne Simmenroth
