# Supplementary material for: Increased professionalization and lower burnout scores were associated with structured residency training program: results of a cross sectional survey
Source: Med Educ Online. 2021 Jul 29;26(1):1959284. doi: 10.1080/10872981.2021.1959284 (PMC8330728; doi:10.1080/10872981.2021.1959284)
Supplement: Supplemental Material [file ZMEO_A_1959284_SM6910.docx]

**Supplement**

**Sensitivity analyses and Pro-D questionnaire**

Study title: Increased professionalization and lower burnout scores were associated with structured residency training program: results of a cross sectional survey

Authors: Michaela Olm, Marco Roos, Alexander Hapfelmeier, Dagmar Schneider, Jochen Gensichen, Pascal O. Berberat, Antonius Schneider

Due to a transmission error, the postal survey (N = 58) of the Maslach Burnout Inventory was only conducted with a 6-point scale (missing category 5: ’A few times a week’). To achieve an approximation of online and postal results, answers in category 4 of the postal questionnaires were replaced by the mean values of 4 and 5 of the online questionnaires. As a result, the adjusted scores of the postal questionnaires showed slightly lower than the scores of the online questionnaires.

The following tables present the sensitivity analyses without the postal questionnaires and only consider the ´online participants´ with correct 7-point Likert scale. The mean values of emotional exhaustion and depersonalization of the ´online group´ were slightly higher, and personal accomplishment slightly lower than for the whole group. Beyond that, there were significant associations between ´year of training´ and professionalism towards other professionals (p = 0.021), and professionalism towards oneself (p = 0.009) (Table 3). The association between ´RTN participation and personal accomplishment´ was higher for the ´online group´ (p < 0.01) than for the whole group (p < 0.05).

**Table S 1** Characteristics of the ambulatory residents (only online participants are presented)

| Total sample (N, (%)) | | 139 | (100) |
| --- | --- | --- | --- |
| Gender: female (N,( %)) | | 113 | (81.3) |
| Age (M, (SD)) | | 37.5 | (7.2) |
| Participation in a regional RTN (N, (%)) | | 28 | (20.1) |
| Working hours per week (M, (SD)) | | 32.9 | (8.4) |
| Year of training | |  |  |
| 1. |  | 6 | (4.3) |
| 2. |  | 15 | (10.8) |
| 3. |  | 21 | (15.1) |
| 4. |  | 49 | (35.3) |
| ≥5. |  | 43 | (30.9) |
| Degree of burnout | |  |  |
| Emotional Exhaustion (N, (%)) | |  |  |
| Low |  | 73 | (52.5) |
| Average |  | 35 | (25.2) |
| High |  | 29 | (20.9) |
| Depersonalization (N, (%)) | |  |  |
| Low |  | 77 | (55.4) |
| Average |  | 26 | (18.7) |
| High |  | 35 | (25.2) |
| Personal Accomplishment (N, (%)) | |  |  |
| Low |  | 88 | (63.3) |
| Average |  | 29 | (20.9) |
| High |  | 12 | (8.6) |

M: mean; N: number; RTN: residents training network; SD: standard deviation.

**Table S 2** Degree of burnout symptoms (mean values of emotional exhaustion, depersonalization, personal accomplishment), differentiated by gender, participation in a regional RTN, training phase, and year of training; only online participants are presented

|  | **Emotional exhaustion** | | | **Depersonalization** | | | **Personal accomplishment** | | |
| --- | --- | --- | --- | --- | --- | --- | --- | --- | --- |
|  | **N** | **M** | **(SD)** | **N** | **M** | **(SD)** | **N** | **M** | **(SD)** |
| **Total** | 137 | 19.3 | (10.0) | 138 | 6.4 | (5.3) | 129 | 41.1 | (5.5) |
| *Gender*^a^ |  |  | |  |  | |  |  | |
| Female | 111 | 19.1 | (9.9) | 112 | 5.9 | (4.9) | 104 | 41.0 | (5.6) |
| Male | 24 | 19.6 | (10.9) | 24 | 8.5 | (6.2) | 23 | 41.3 | (5.2) |
|  |  | *p = 0.811* | |  | *p = 0.032* | |  | *p = 0.990* | |
| *Year of training*^b^ |  |  | |  |  | |  |  | |
| 1. | 5 | 28.8 | (7.6) | 6 | 8.3 | (7.4) | 4 | 36.5 | (5.2) |
| 2. | 15 | 17.1 | (8.6) | 15 | 6.4 | (5.2) | 13 | 40.6 | (4.6) |
| 3. | 21 | 17.9 | (10.3) | 21 | 6.6 | (5.7) | 20 | 41.5 | (5.3) |
| 4. | 48 | 19.1 | (9.7) | 49 | 5.9 | (4.6) | 47 | 41.9 | (4.2) |
| ≥5. | 43 | 20.3 | (11.1) | 42 | 6.4 | (5.8) | 41 | 40.5 | (7.0) |
|  |  | *p = 0.167* | |  | *p = 0.976* | |  | *p = 0.405* | |
| *Participation in a regional RTN*^a^ | | | |  |  | |  |  | |
| Yes | 28 | 21.4 | (12.9) | 28 | 7.0 | (6.4) | 25 | 38.8 | (7.3) |
| No | 100 | 19.0 | (9.2) | 101 | 6.4 | (5.1) | 96 | 41.7 | (4.9) |
|  |  | *p = 0.487* | |  | *p = 0.973* | |  | *p = 0.092* | |

^a^Test of significance: Mann-Whitney U-test; ^b^Test of significance: Kruskal-Wallis-test; Level of significance: p < 0.05; M: mean; N: number; RTN: residents training network; SD: standard deviation. Cut-off values for ‘high’ burnout as suggested by Maslach et al.[18]: emotional exhaustion: ≥27; depersonalization: ≥10; personal accomplishment: ≤33.

**Table S 3** Degree of professionalism (sum score), differentiated by gender, participation in a regional network, and year of training; only online participants are presented

|  | **Professionalism towards the patient** | | | **Professionalism towards other professionals** | | | **Professionalism towards society** | | | **Professionalism towards oneself** | | |
| --- | --- | --- | --- | --- | --- | --- | --- | --- | --- | --- | --- | --- |
|  | **N** | **M** | **(SD)** | **N** | **M** | **(SD)** | **N** | **M** | **(SD)** | **N** | **M** | **(SD)** |
| **Total** | 139 | 72.5 | (5.5) | 119 | 44.2 | (5.4) | 129 | 31.3 | (4.3) | 138 | 71.4 | (6.7) |
| *Participation in a regional RTN*^a^ | | | | |  | |  |  | |  |  | |
| Yes | 28 | 74.9 | (5.0) | 25 | 46.3 | (4.3) | 27 | 33.3 | (4.1) | 28 | 73.0 | (7.7) |
| No | 102 | 72.0 | (5.4) | 86 | 43.7 | (5.6) | 93 | 30.7 | (4.2) | 101 | 71.0 | (6.6) |
|  |  | *p = 0.014* | |  | *p = 0.023* | |  | *p = 0.007* | |  | *p = 0.192* | |
| *Gender*^a^ | |  | |  |  | |  |  | |  |  | |
| Female | 113 | 73.0 | (5.3) | 96 | 44.4 | (5.5) | 105 | 31.2 | (4.4) | 112 | 71.9 | (6.3) |
| Male | 24 | 69.9 | (5.6) | 21 | 43.0 | (5.2) | 22 | 31.6 | (4.0) | 24 | 69.3 | (8.4) |
|  |  | *p = 0.019* | |  | *p = 0.289* | |  | *p = 0.709* | |  | *p = 0.276* | |
| *Year of training* | | | | |  | |  |  | |  |  | |
| 1. | 6 | 68.1 | (3.5) | 5 | 41.4 | (3.2) | 5 | 30.9 | (2.5) | 5 | 64.4 | (5.9) |
| 2. | 15 | 71.1 | (5.2) | 10 | 42.6 | (5.9) | 13 | 30.1 | (5.4) | 15 | 69.6 | (5.9) |
| 3. | 21 | 71.9 | (6.2) | 20 | 44.0 | (5.0) | 19 | 30.8 | (4.0) | 21 | 72.5 | (5.8) |
| 4. | 49 | 72.6 | (5.2) | 41 | 43.3 | (5.0) | 46 | 31.1 | (4.2) | 49 | 70.9 | (7.0) |
| ≥5. | 43 | 74.1 | (4.9) | 38 | 46.7 | (5.5) | 41 | 32.7 | (4.0) | 43 | 73.4 | (6.0) |
|  |  | *p = 0.049* | |  | *p = 0.021* | |  | *p = 0.246* | |  | *p = 0.009* | |

^a^Test of significance: Mann-Whitney U-test; ^b^Test of significance: Kruskal-Wallis-test; Level of significance: p < 0.05; M: mean; N: number; RTN: residents training network; SD: standard deviation.

**Table S 4** Multivariable linear regression modelling of professionalism, year of training, and participation in a regional RTN. Outcome: Burnout sum scores of emotional exhaustion, depersonalization, and personal accomplishment; only online participants are presented

| **Explanatory variable** | **Emotional exhaustion** | | | **Depersonalization** | | | **Personal accomplishment** | | |
| --- | --- | --- | --- | --- | --- | --- | --- | --- | --- |
|  | **β** | **SD** | **p value** | **β** | **SD** | **p value** | **β** | **SD** | **p value** |
| Constant | 58.066 | 14.318 |  | 30.691 | 7.360 |  | 13.220 | 7.345 |  |
| Professionalism towards the patient | -0.470 | 0.182 | 0.011 | -0.317 | 0.094 | -0.309 | 0.456 | 0.093 | 0.000 |
| Year of training | 0.484 | 0.839 | 0.565 | 0.242 | 0.428 | 0.051 | -0.200 | 0.440 | 0.650 |
| Participation in a regional RTN | -3.332 | 2.245 | 0.140 | -1.175 | 1.159 | -0.090 | 4.025 | 1.163 | 0.001 |
| Constant | 40.282 | 10.716 |  | 16.589 | 5.616 |  | 20.818 | 5.531 |  |
| Professionalism towards other professionals | -0.392 | 0.200 | 0.053 | -0.206 | 0.105 | 0.051 | 0.302 | 0.100 | 0.003 |
| Year of training | 0.354 | 0.957 | 0.712 | -0.047 | 0.501 | 0.926 | -0.321 | 0.497 | 0.643 |
| Participation in a regional RTN | -2.529 | 2.505 | 0.315 | -0.435 | 1.308 | 0.740 | 4.385 | 1.287 | 0.001 |
| Constant | 38.581 | 9.530 |  | 14.481 | 4.983 |  | 25.601 | 4.894 |  |
| Professionalism towards society | -0.473 | 0.236 | 0.047 | -0.216 | 0.123 | 0.083 | 0.284 | 0.120 | 0.020 |
| Year of training | 0.328 | 0.884 | 0.711 | -0.012 | 0.465 | 0.980 | -0.089 | 0.472 | 0.852 |
| Participation in a regional RTN | -3.042 | 2.373 | 0.202 | -0.759 | 1.241 | 0.542 | 4.013 | 1.241 | 0.002 |
| Constant | 58.040 | 10.733 |  | 29.183 | 5.504 |  | 10.569 | 5.550 |  |
| Professionalism towards oneself | -0.498 | 0.137 | 0.000 | -0.305 | 0.070 | 0.000 | 0.354 | 0.070 | 0.000 |
| Year of training | 0.667 | 0.818 | 0.417 | 0.149 | 0.422 | 0.725 | -0.218 | 0.439 | 0.621 |
| Participation in a regional RTN | -2.935 | 2.155 | 0.176 | -0.824 | 1.108 | 0.459 | 3.359 | 1.139 | 0.004 |

RTN: residents training network; SD: standard deviation; Level of significance: p < 0.05; Note: Higher values in emotional exhaustion and depersonalization, but lower values in personal accomplishment indicate a higher degree of burnout symptoms.

**Table S 5** Pro-D (German Professionalism Scale) questionnaire; English version

| Item | *The family medicine resident…* |  |  |  |  |
| --- | --- | --- | --- | --- | --- |
| Domain: professionalism towards the patient | | seldom | sometimes | often | always |
| 1.1 | *Deals correctly with legislative rules regarding informed consent* | ○ | ○ | ○ | ○ |
| 1.2 | *Is able to bring up difficult subjects* | ○ | ○ | ○ | ○ |
| 1.3 | *Respects the right of patients to inspect their medical records* | ○ | ○ | ○ | ○ |
| 1.4 | *Is able to show sympathy* | ○ | ○ | ○ | ○ |
| 1.5 | *Takes patients’ embarrassment, shyness and reluctance into account* | ○ | ○ | ○ | ○ |
| 1.6 | *During physical examinations, explains the aim of the procedures and what is expected of the patient* | ○ | ○ | ○ | ○ |
| 1.7 | *Approaches patients with a different frame of reference (e.g. religion) openly* | ○ | ○ | ○ | ○ |
| 1.8 | *Looks clean and tidy and dresses according to current norms* | ○ | ○ | ○ | ○ |
| 1.9 | *Adjusts language to communicate with patients* | ○ | ○ | ○ | ○ |
| 1.10 | *Takes sex specific differences into account* | ○ | ○ | ○ | ○ |
| 1.11 | *Is able to cope with the different expectations that patients have of their GP* | ○ | ○ | ○ | ○ |
| 1.12 | *Involves the previous history of the patient in the provision of care* | ○ | ○ | ○ | ○ |
| 1.13 | *Pays attention to the consequence of treatment policy on the daily functioning of the patient* | ○ | ○ | ○ | ○ |
| 1.14 | *Involves relevant aspects of the patient’s home and environment in the provision of care* | ○ | ○ | ○ | ○ |
| 1.15 | *Retains insight into the medical history of the patients in order to act proactively if necessary* | ○ | ○ | ○ | ○ |
| 1.16 | *If necessary, takes action after life events* | ○ | ○ | ○ | ○ |
| 1.17 | *Respects patients’ self-determination* | ○ | ○ | ○ | ○ |
| 1.18 | *Deals carefully with professional secrecy when talking to colleagues or acquaintances* | ○ | ○ | ○ | ○ |
| 1.19 | *Does not give patients false hope* | ○ | ○ | ○ | ○ |
| 1.20 | *Takes care not to become too involved in the patient’s emotions* | ○ | ○ | ○ | ○ |
| 1.21 | *Takes care not to be influenced by patients of high social status* | ○ | ○ | ○ | ○ |
| Domain: professionalism towards other professionals | |  |  |  |  |
| 2.1 | *Consults other care providers with targeted questions* | ○ | ○ | ○ | ○ |
| 2.2 | *Ensures structured information transfer with other care providers* | ○ | ○ | ○ | ○ |

*Continue of Table S 5*

| Item | *The family medicine resident…* | seldom | sometimes | often | always |
| --- | --- | --- | --- | --- | --- |
| 2.3 | *Deals correctly with targeted questions from other care providers* | ○ | ○ | ○ | ○ |
| 2.4 | *Is able to motivate support personnel* | ○ | ○ | ○ | ○ |
| 2.5 | *Makes clear agreements with support personnel* | ○ | ○ | ○ | ○ |
| 2.6 | *Listens to the contributions of support personnel* | ○ | ○ | ○ | ○ |
| 2.7 | *Transfers services correctly* | ○ | ○ | ○ | ○ |
| 2.8 | *Discusses bottlenecks in cooperation with others directly* | ○ | ○ | ○ | ○ |
| 2.9 | *Is able to deal constructively with conflicts* | ○ | ○ | ○ | ○ |
| 2.10 | *Is able to manage the mutual demarcation of tasks between GP and specialists* | ○ | ○ | ○ | ○ |
| 2.11 | *Ensures coherence in first and second line medical care* | ○ | ○ | ○ | ○ |
| 2.12 | *Is able to distinguish between professional and personal interests in negotiations* | ○ | ○ | ○ | ○ |
| 2.13 | *Is able to take policy decisions* | ○ | ○ | ○ | ○ |
| 2.14 | *Is able to conduct job evaluations* | ○ | ○ | ○ | ○ |
| Domain: professionalism towards society | |  |  |  |  |
| 3.1 | *Bears the consequences of his/her own conduct* | ○ | ○ | ○ | ○ |
| 3.2 | *Is able to justify deviations from rules and guidelines* | ○ | ○ | ○ | ○ |
| 3.3 | *Is aware of his/her own norms regarding disease influence disease management* | ○ | ○ | ○ | ○ |
| 3.4 | *Is aware of the meaning and the relative value of scientific evidence in decision-making* | ○ | ○ | ○ | ○ |
| 3.5 | *In decision-making, weighs scientific evidence against factors related to the patient or the circumstances* | ○ | ○ | ○ | ○ |
| 3.6 | *Is able to justify choices made on the basis of scientific evidence* | ○ | ○ | ○ | ○ |
| 3.7 | *Is able to explain his/her own norms and values regarding the application of scientific evidence* | ○ | ○ | ○ | ○ |
| 3.8 | *Is able to estimate which problems are suitable for a quality-improvement project* | ○ | ○ | ○ | ○ |
| 3.9 | *Is able to work out a quality-improvement project* | ○ | ○ | ○ | ○ |
| 3.10 | *Is able to justify indications for making home visits* | ○ | ○ | ○ | ○ |

*Continue of Table S 5*

| Item | *The family medicine resident…* |  |  |  |  |
| --- | --- | --- | --- | --- | --- |
| Domain: professionalism towards oneself | | seldom | sometimes | often | always |
| 4.1 | *Is able to name reactions, thoughts and feelings that patients evoke* | ○ | ○ | ○ | ○ |
| 4.2 | *Asks questions about his/her own role in relationships (patient, team, family physician, trainer, etc.)* | ○ | ○ | ○ | ○ |
| 4.3 | *Uses specific practical situations as starting points for critical self-reflection* | ○ | ○ | ○ | ○ |
| 4.4 | *Discusses his/her own shortcomings and failures without losing belief in his/her own competence* | ○ | ○ | ○ | ○ |
| 4.5 | *Makes a realistic estimation of his/her own strong and weak points* | ○ | ○ | ○ | ○ |
| 4.6 | *Is able to balance work and private life* | ○ | ○ | ○ | ○ |
| 4.7 | *Is able to mention aspects of work that increase satisfaction* | ○ | ○ | ○ | ○ |
| 4.8 | *Is able to deal with the possibility that a treatment decision may be unsuccessful* | ○ | ○ | ○ | ○ |
| 4.9 | *Adheres to agreements made during feedback* | ○ | ○ | ○ | ○ |
| 4.10 | *Attaches importance to what others think about his/her behaviour* | ○ | ○ | ○ | ○ |
| 4.11 | *Does not resists being judged* | ○ | ○ | ○ | ○ |
| 4.12 | *Has an enquiring mind (asks questions and takes initiatives)* | ○ | ○ | ○ | ○ |
| 4.13 | *Is able to admit his/her own mistakes* | ○ | ○ | ○ | ○ |
| 4.14 | *Takes action to rectify his/her own mistakes* | ○ | ○ | ○ | ○ |
| 4.15 | *Withdraws from the consequences of his/her own mistakes* | ○ | ○ | ○ | ○ |
| 4.16 | *Is able to adapt and keep control of the situation if patients unexpectedly need to be seen during other activities* | ○ | ○ | ○ | ○ |
| 4.17 | *Recovers rapidly after an unpleasant consultation* | ○ | ○ | ○ | ○ |
| 4.18 | *Is able to let a mild disorder (e.g. tiredness) run its own course even though the correct diagnosis is a mystery* | ○ | ○ | ○ | ○ |
| 4.19 | *Is able to cope after making a mistake* | ○ | ○ | ○ | ○ |
| 4.20 | *Is able to deal with difficult or angry patients* | ○ | ○ | ○ | ○ |
| 4.21 | *Is able to conduct interventions that lead to decrease in aggression from the patient* | ○ | ○ | ○ | ○ |

Original questionnaire (German version) can be found in: Roos M, Pfisterer D, Krug D, Ledig T, Steinhäuser J, Szecsenyi J, et al. Adaptation, psychometric properties and feasibility of the Professionalism Scale Germany. Zeitschrift fur Evidenz, Fortbildung und Qualitat im Gesundheitswesen. 2016;113:66-75.
